# Modulation of recombination zone position for quasi-two-dimensional blue perovskite light-emitting diodes with efficiency exceeding 5%

**DOI:** 10.1038/s41467-019-09011-5

**Published:** 2019-03-04

**Authors:** Zhenchao Li, Ziming Chen, Yongchao Yang, Qifan Xue, Hin-Lap Yip, Yong Cao

**Affiliations:** State Key Laboratory of Luminescent Materials and Devices, Institute of Polymer Optoelectronic Materials and Devices, School of Materials Science and Engineering, South China University of Technology, 381 Wushan Road, 510640 Guangzhou, China

## Abstract

In recent years, substantial progress has been made in developing perovskite light-emitting diodes with near-infrared, red and green emissions and over 20% external quantum efficiency. However, the development of perovskite light-emitting diodes with blue emission remains a great challenge, which retards further development of full-color displays and white-light illumination based on perovskite emissive materials. Here, firstly, through composition and dimensional engineering, we prepare quasi-two-dimensional perovskite thin films with improved blue emission, taking advantages of reduced trap density and enhanced photoluminescence quantum yield. Secondly, we find a vertically non-uniform distribution of perovskite crystals in the PEDOT:PSS/perovskite hybrid film. Through modulating the position of the recombination zone, we activate the majority of quasi-two-dimensional perovskite crystals, and thus demonstrate the most efficient blue perovskite light-emitting diode to date with emission peak at 480 nm, record luminance of 3780 cd m^−2^ and record external quantum efficiency of 5.7%.

## Abstract (web summary)

Halide perovskite based light-emitting diodes attracted intensive research interest recently but the efficiency of blue diodes is much lower than the green and red ones. Here Li et al. push up the efficiency of blue diodes through composition engineering and vertical morphology control.

## Introduction

In recent years, metal halide perovskites have shown promising potential in a series of optoelectronic applications such as solar cells, light-emitting diodes (LEDs), photodetectors, and lasers^1–4^. The booming research on metal halide perovskites can be attributed to their superior optoelectronic properties such as high absorption coefficient, long charge-carrier diffusion length, high photoluminescence quantum yield (PLQY), and high defect tolerance^5–8^. In particular, owing to the nature of their facile color tunability, solution processability, and sharp emission, metal halide perovskites have been successfully applied in LEDs, extending the family of emitters^9–15^. Substantial progress has been made over the past several years in perovskite LEDs by modulating the microstructures of the perovskite emitter, suppressing surface defects, and utilizing dimensional engineering. Based on these strategies, perovskite LEDs with near-infrared, red, and green emissions have reached over 20% external quantum efficiency (EQE)^16–18^. However, compared with the efficient near-infrared, red, and green perovskite LEDs, blue perovskite LEDs currently still suffer from inferior performance. Improving performance remains a great challenge and retards the development of possible applications such as full-color displays and white-light illumination based on perovskite LEDs. Therefore, a breakthrough in thin film perovskite LEDs with blue emission is an urgent target in this field.

As blue emissions are generally considered to be in the range of ~450–500 nm, from deep blue to sky blue, in perovskite thin film emitters, there are typically two approaches that can achieve emission in this range: dimensional engineering and composition engineering. In terms of dimensional engineering, a hypsochromic shift of emission can be obtained by quantum confinement effect when reducing the dimension of perovskite from three dimensions to two dimensions. Generally, the blue photoluminescence (PL) and electroluminescence (EL) of perovskite thin films and LEDs can be achieved by forming quasi-two-dimensional (quasi-2D) perovskite from a three-dimensional (3D) bulk perovskite. In our previous work, we incorporated a small molecule of 2-phenoxyethylamine into CH_3_NH_3_PbBr_3_ to form 2D perovskite to blue shift the EL from 532 to 494 nm and 462 nm to achieve the sky-blue and deep-blue perovskite LEDs, respectively^19^. A similar strategy was applied by using ethylammonium bromide as the ligand to form 2D perovskite thin films, which yielded a decent EQE of 2.6% with EL peaks of 473 and 485 nm^20^. To the best of our knowledge, this performance is the leading result among all of the published data. However, the relatively poor thermal stability and ion migration problems existing in organic–inorganic hybrid perovskite are detrimental to device stability, particularly in blue perovskite LEDs, which require a higher working voltage and hence stronger electric field for operation^21,22^. Therefore, a much more stable perovskite system is required for blue perovskite LEDs. Among the perovskite family, an all-inorganic perovskite system obtains much better structural and thermal stability than an organic–inorganic hybrid system^23,24^. Hence, it is possible to achieve a more stable blue perovskite LED by forming a quasi-2D perovskite from a green-emissive all-inorganic 3D perovskite of CsPbBr_3_. According to the previous reports and our own case (Supplementary Figure 1a), the formation of quasi-2D perovskite could indeed blue shift the PL from the green to blue region by the quantum confinement effect^19,25^. Unfortunately, in the case of perovskite LEDs based on these quasi-2D perovskite films, all the high energy blue emissions diminished and only the green-emissive EL was detected (Supplementary Figure 1b), and similar phenomenon was also observed in other report^25^. We attribute this phenomenon to the highly efficient energy transfer and/or charge transfer from larger bandgap perovskite to the smallest bandgap perovskite in such an all-inorganic quasi-2D perovskite system, as has also been observed in other reports and was used as a key strategy to improve the emission property of small bandgap perovskites^26,27^. The failure in achieving blue perovskite LED by forming quasi-2D perovskite based on a CsPbBr_3_ system prompts us to enlarge the bandgap of the 3D component in quasi-2D perovskite films to obtain a blue EL regardless of the presence of highly efficient energy transfer and/or charge transfer from the 2D to 3D component.

Composition engineering on halide anions is a proven strategy to tune the bandgap of 3D perovskite. It is well-known that partially replacing Br with Cl in an AMBr_3_ perovskite system (where A represents monovalent cations and M represents metal cations) can enlarge the bandgap of perovskite and hence blue shift its emission from green to blue^28^. Here we show the perovskite of CsPbCl_0.9_Br_2.1_ with emission at 484 nm in the blue region through composition engineering by incorporating Cl into CsPbBr_3_ lattice. Based on such a 3D perovskite system, phenylethylammonium bromide (PEABr) is subsequently introduced into the CsPbCl_0.9_Br_2.1_ perovskite to form a quasi-2D perovskite thin film. We find that the PEABr can efficiently passivate the traps in perovskite thin films and dramatically improve their PLQYs from 0.15% to a maximum of 27%. Moreover, we observe a vertically non-uniform distribution of perovskite crystals in the Poly(3,4-ethylenedioxythiophene):poly(styrene sulfonate) (PEDOT:PSS)/perovskite hybrid film in which the majority of perovskite crystals disperse at the top of the film. On the basis of the recombination zone control in this hybrid layer, we activate most of the perovskite emitters and thus successfully demonstrate a highly efficient blue perovskite LED with maximum EQE of 5.7% and maximum luminance of 3780 cd m^−2^, which, to our best knowledge, is a record performance for perovskite LEDs with blue emission.

## Results

### Optical properties of composition-engineered perovskites

**Fig. 1:**
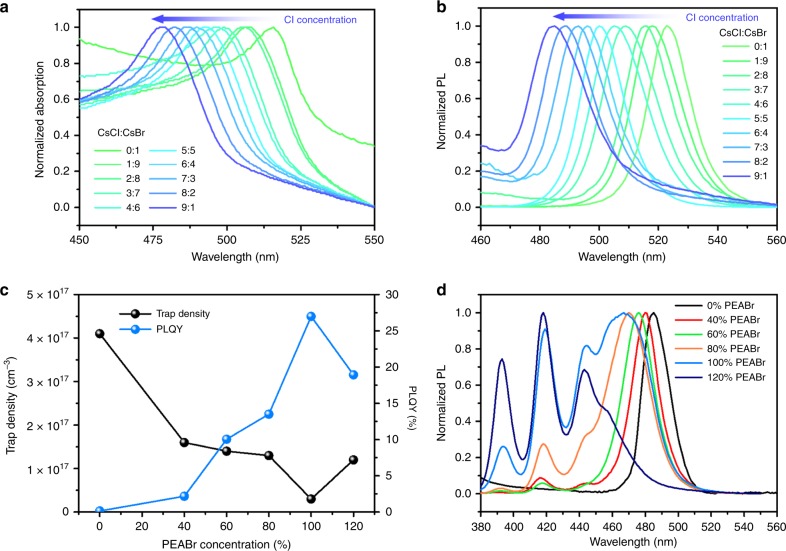
Composition engineering for efficient blue emission of perovskite. **a** Normalized absorption of CsPbCl_*x*_Br_3 − *x*_ thin films with different molar ratios of cesium chloride (CsCl) to cesium bromide (CsBr). The results are normalized by the intensity of their corresponding excitonic absorption peaks. **b** Photoluminescence (PL) spectra of CsPbCl_*x*_Br_3 − *x*_ thin films with different molar ratios of CsCl to CsBr. **c** Trap densities and photoluminescence quantum yields (PLQYs) of CsPbCl_0.9_Br_2.1_ thin films with different ratios of phenylethylammonium bromide (PEABr). The trap density was calculated based on the equation *N*_t_ = 2*ε*_0_*ε*_r_*V*_TFL_/(*qL*^2^), and a detailed discussion can be found in Supplementary Figures 3 and 4. **d** PL spectra of CsPbCl_0.9_Br_2.1_ thin films with different ratios of PEABr

Typically, all inorganic CsPbBr_3_ perovskite, which emits green light, is composed of cesium bromide (CsBr) and lead bromide (PbBr_2_). As previously mentioned, the incorporation of Cl into the CsPbBr_3_ lattice makes possible the shift in emission from green to blue. However, the extremely poor solubility of CsPbCl_3_ (which is composed of cesium chloride (CsCl) and PbCl_2_) in dimethyl sulfoxide (DMSO) limits the Cl concentration in the CsPbCl_*x*_Br_3*−* *x*_ precursor solution and hence limits the degree of hypsochromic shift of emission. Compared to PbCl_2_, CsCl has a better solubility in DMSO, which can be applied as one of the precursor materials for CsPbCl_*x*_Br_3 − *x*_ to enhance its Cl concentration. Therefore, CsCl, CsBr, and PbBr_2_ are used to form CsPbCl_*x*_Br_3 − *x*_ in our case. We studied the different molar ratios between CsCl and CsBr and the total molar ratio between (CsCl + CsBr) and PbBr_2_ is fixed to a 1:1 ratio in CsPbCl_*x*_Br_3 − *x*_ precursor solution. According to Fig. 1, as we increased the Cl concentration in CsPbCl_*x*_Br_3 − *x*_ by carefully increasing the amount of CsCl, a gradual hypsochromic shift of absorption edges from green to blue was observed, illustrating that we could precisely modulate the emission color for CsPbCl_*x*_Br_3 − *x*_ perovskite. When the ratio of CsCl to CsBr reached 9:1, the absorption peak of CsPbCl_0.9_Br_2.1_ perovskite appeared at 478 nm in the blue region. However, when we further increased the ratio to 1:0, precipitation occurred in the precursor solution (Supplementary Figure 2), suggesting that the precursor solution reached a solubility limit and became unsuitable for further processing into thin films. According to Fig. 1b, a similar trend was also observed in the PL spectra. When we increased the Cl concentration in the CsPbCl_*x*_Br_3 −__ *x*_ lattice, a gradual hypsochromic shift of emission was obtained, and the bluest emission, which was achieved by the CsPbCl_0.9_Br_2.1_ perovskite, was located at the wavelength of 484 nm in the blue region. Therefore, the composition of CsPbCl_0.9_Br_2.1_ was selected as the 3D perovskite system for our subsequent study aiming to realize the target of an efficient blue perovskite LED.

In metal halide perovskites, trap-assisted recombination, which relates to trap density in perovskite thin films, is a typically non-radiative recombination^2^. According to Fig. 1c, the trap density in a pristine CsPbCl_0.9_Br_2.1_ perovskite thin film is around 4.1 × 10^17^ cm^−3^, causing a low PLQY of around 0.15%. Noticing that the charge-carrier density in a working LED is generally of the order of <10^16^ cm^−3^, it can be expected that such high trap density in pristine CsPbCl_0.9_Br_2.1_ perovskite thin film will probably lead to inferior performance of the corresponding LED device^29^. Therefore, the suppression of trap-assisted recombination by reducing the trap density is critical to achieving a higher PLQY and better device performance. Recent reports prove that 2D perovskite obtains a much lower trap density compared with its 3D counterpart because the organic ligands (such as 2­phenoxyethylamine and butylammonium) generally passivate surface traps of perovskite^19,30^. With this inspiration, we used PEABr to form 2D perovskite and passivated its surface traps based on the CsPbCl_0.9_Br_2.1_ system. Encouragingly, the trap density of perovskite thin films indeed dramatically reduced from ~4.1 × 10^17^ to 3.0 × 10^16^ cm^−3^ as the ratio of PEABr was increased from 0 to 100% (Supplementary Figures 3 and 4). However, a further increase in PEABr concentration to 120% resulted in an increase of trap density to around 1.2 × 10^17^ cm^−3^. This result was attributed to the reduced crystal quality in perovskite films (which will be discussed later). In addition, we performed PL lifetime measurement to cross-check the trap passivation effect. The PL lifetimes of perovskite films increased as the increase of PEABr ratios (Supplementary Figure 5), which suggested that the surface trap states of perovskite had been well passivated by the PEABr molecules. Furthermore, from Fig. 1c, we can observe that the PLQY is increased with reducing the trap density of the perovskite film. The PLQYs of the perovskite thin films dramatically increase from 0.15 to 27% with the increase of PEABr ratios from 0 to 100%, and it decreases again to 18.9% when an overload of 120% PEABr was added into the CsPbCl_0.9_Br_2.1_ system. The relationship between trap densities and PLQYs of perovskite thin films shows that trap-assisted recombination dominates the non-radiative recombination, and trap reduction in such mixed-halide perovskites is critical to achieving efficient blue emission.

We also recorded the PL spectra of CsPbCl_0.9_Br_2.1_ thin films with different ratios of PEABr to reveal their emission colors. Interestingly, as the PEABr ratio increased, the emission peaks gradually blue shifted, and several specific peaks appeared in the deep-blue region. Similar to the CsPbBr_3_ system discussed previously, the presence of excitonic emission peaks at 393, 418, 444, and 457 nm reflected the formation of 2D PEA_2_Cs_*n* − 1_Pb_*n*_(Cl_*x*_Br_1 − *x*_)_3*n* + 1_ perovskite (corresponding to *n* = 1, 2, 3, and 4, respectively). Therefore, 2D perovskites with *n* = 1, 2, and 3 coexisted in CsPbCl_0.9_Br_2.1_ thin films with 40% PEABr, while the CsPbCl_0.9_Br_2.1_ thin films with 60%, 80%, 100%, and 120% PEABr contained 2D perovskites with *n* values of 1, 2, 3, and 4. In addition, the resultant films are quasi-2D perovskite films composed of mixture of both 2D and 3D phases as indicated by the coexistence of characteristic emission at lower energy from the 3D component.

### Nanostructure and morphology of perovskite thin films

**Fig. 2:**
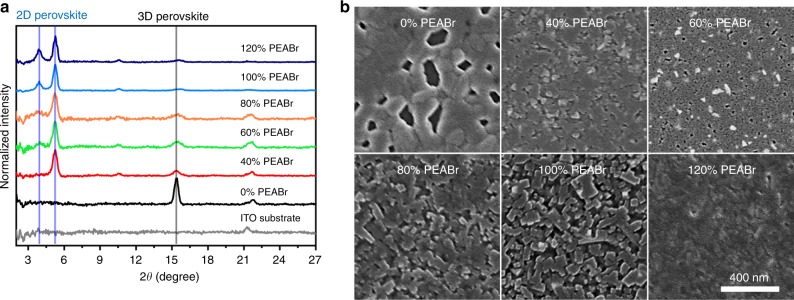
Crystal structure and morphology of CsPbCl_0.9_Br_2.1_ thin films with different ratios of phenylethylammonium bromide (PEABr). **a** X-ray diffraction (XRD) patterns and **b** top-view scanning electron microscope (SEM) images at a size of 1 × 1 μm^2^

To further confirm the coexistence of 2D and 3D perovskites in the film, we used X-ray diffraction (XRD) measurement to study the structural property of the perovskite films. According to Fig. 2a, in pristine CsPbCl_0.9_Br_2.1_ thin film, a main peak at 2*θ* of 15.4° appeared, corresponding to the (100) plane of 3D perovskite. When we added 40% PEABr into CsPbCl_0.9_Br_2.1_ thin films, a specific peak at 2*θ* of 5.3° appeared, reflecting the formation of *n* = 1 2D perovskite^31^. When we further increased the PEABr ratio to over 60%, another specific peak at 2*θ* of 3.9° appeared, and we accordingly assigned it to the *n* = 2 2D perovskite. According to previous report that when PEABr was used as the organic spacer to form 2D perovskite based on Cs_*x*_MA_1 − *x*_PbBr_3_ system, the *n* = 1 2D perovskite obtained the lowest formation energy than that in the 2D perovskites with higher *n*, which might explain the formation of *n* = 1 2D perovskite even though the PEABr ratio was low^32^. In addition, although we dramatically increased the PEABr ratio from 0 to 120%, the XRD pattern from 3D perovskite was always present, also suggesting that the film we obtained was a quasi-2D perovskite film that combined with 2D and 3D perovskites. It is noteworthy that based on PL measurement, several specific emission peaks from different types of 2D perovskite (*n* = 1, 2, 3, and 4) appeared, whereas only *n* = 1 and/or *n* = 2 perovskites were detected in the XRD measurements. We attributed this mismatch to two possible reasons: first, the orientations of 2D perovskite crystals with higher *n* value are not in out-of-plane direction, which become undetectable in the one-dimensional XRD measurement; second, the non-periodic alignment of these 2D perovskite crystals with higher *n* value caused the absence of Bragg diffraction and hence the absence of an XRD signal^33^.

The morphology of perovskite thin films is another important factor governing the final performance of perovskite LEDs. Therefore, we used a scanning electron microscope (SEM) to characterize the morphology of perovskite thin films with different ratios of PEABr. Figure 2b shows that the pristine CsPbCl_0.9_Br_2.1_ film has a poor morphology with a large number of pinholes. However, after the introduction of PEABr, perovskite films with better morphology and coverage were obtained. As the PEABr ratio increased from 40 to 100%, the perovskite crystal became more and more regular in shape, suggesting improved crystal quality of the perovskite thin film. Particularly in the 100% PEABr case, a perovskite film with isolated and regular perovskite small crystals was obtained, reflecting the formation of a high-quality perovskite film that correlated well with its low trap density and high PLQY. However, when we further increased the PEABr ratio to 120%, the perovskite crystal returned to an irregular shape, resulting in a perovskite film with poorer crystal quality. It is likely that too much PEABr suppressed the formation of perovskite crystals, with a similar phenomenon also being observed when perovskite was mixed with other amine-containing materials^16,34^.

### Device structure and performance

**Fig. 3:**
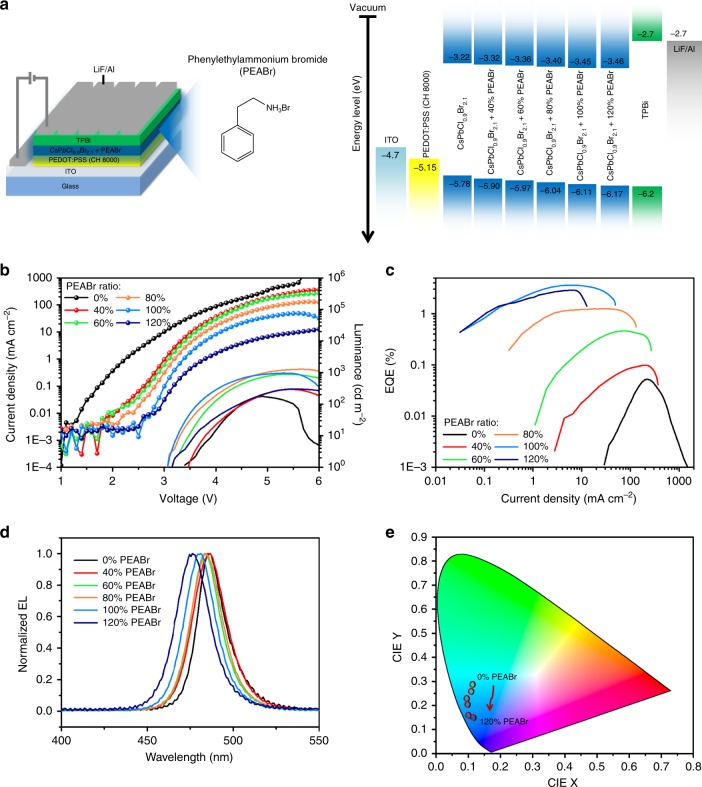
Performance of CsPbCl_0.9_Br_2.1_ perovskite light-emitting diodes (LEDs) with different ratios of phenylethylammonium bromide (PEABr). **a** Perovskite LED architecture and energy level diagram for different layers of the device. **b** Characterization of current density and luminance versus voltage. The solid lines and lines with dots correspond to the luminance and the current density, respectively. **c** Characterization of external quantum efficiency (EQE) versus current density. **d** Electroluminescence (EL) spectra of perovskite LEDs. **e** Commission Internationale de l’Eclairage (CIE) values of the EL spectra of perovskite LEDs

To evaluate the EL properties of CsPbCl_0.9_Br_2.1_ thin films with different ratios of PEABr, we fabricated perovskite LEDs based on the device architecture of indium tin oxide (ITO)/PEDOT:PSS/perovskite/1, 3, 5-tris(*N*-phenylbenzimiazole-2-yl)benzene (TPBi)/lithium fluoride (LiF)/Al, as shown in Fig. 3a. Here, PEDOT:PSS (CH 8000, 45 nm thick) with a deep work function of −5.15 eV was used as the hole injection layer^35^. The valance band of perovskite was confirmed by ultraviolet photoelectron spectroscopy (UPS) as shown (Supplementary Figure 6) and the energy diagram of the whole device is presented in Fig. 3a. We also summarize the device performance in Fig. 3b, c and Table 1. In the pristine CsPbCl_0.9_Br_2.1_ perovskite LED, an inferior EQE of only 0.05% was achieved. When we increased the PEABr ratio in CsPbCl_0.9_Br_2.1_ perovskite thin films from 0 to 100%, the device performance improved dramatically, and the best device reached a high EQE of 3.6%. However, further increasing the PEABr ratio to 120% reduced the EQE and luminance. There are three potential reasons for these results. First, the trend of EQE change with increasing PEABr ratios is similar to that of PLQYs of perovskite films, suggesting that the EL property of perovskite LEDs is governed by the PL property of the corresponding perovskite thin films. Second, the EL property also strongly related to the trap densities of perovskite films as higher trap density could result in more severe trapping of injected charge carriers and hence cause a more severe non-radiative trap-assisted recombination. Third, the pristine perovskite thin film suffered from poor morphology due to the large number of pinholes that caused a severe leakage current in the LED device, while better film coverage and crystal quality were obtained after the introduction of PEABr from 0 to 100%, which also resulted in the suppression of leakage current in perovskite LEDs according to Fig. 3b. However, a further increase in the PEABr concentration to 120% led to a film with decreased crystal quality compared with the 100% PEABr case, causing a slightly drop in device performance.

**Table 1:** Summary of CsPbCl_0.9_Br_2.1_ perovskite LEDs with different ratios of PEABr

PEABr ratio	*V*_T_ (V)	EL peak (nm)	Max. L. (cd m^−2^)	Max. CE (cd A^−1^)	Max. EQE (%)	FWHM (nm)	CIE
0%	3.5	487	177	0.07	0.05	20	(0.112,0.289)
40%	3.5	485	293	0.12	0.10	23	(0.105,0.254)
60%	3.3	484	892	0.52	0.46	21	(0.093,0.222)
80%	3.2	483	1278	1.43	1.25	21	(0.096,0.204)
100%	3.1	480	965	3.90	3.60	21	(0.101,0.155)
120%	3.3	478	304	2.69	2.88	24	(0.113,0.149)

*LED* light-emitting diode, *PEABr* phenylethylammonium bromide, *V*_T_ turn-on voltage, *CE* current efficiency, *EQE* external quantum efficiency, *FWHM* full-width at half-maximum, *CIE* Commission Internationale de l’Eclairage

Figure 3d shows the EL spectra of the CsPbCl_0.9_Br_2.1_ perovskite LEDs with different ratios of PEABr and all the EL emissions obtained narrow full-widths at half-maximum (FWHMs) of <25 nm, resulting in the high color purity in Commission Internationale de l’Eclairage (CIE) coordinate as shown in Fig. 3e. Similar to the CsPbBr_3_ system, although excitonic PL from 2D PEA_2_Cs_*n* − 1_Pb_*n*_(Cl_*x*_Br_1 − *x*_)_3*n* + 1_ perovskites was observed, only one single EL peak at the lowest energy belonging to the 3D perovskite was detected in all cases, suggesting that energy transfer and/or charge transfer from higher-energy 2D perovskite to lower-energy 3D perovskite is also highly efficient in such a quasi-2D system. In addition, a gradual blue shift of EL from 487 to 478 nm was observed as the PEABr ratio increased, which correlated well with the blue shift of PL spectra because of the gradual loss of the 3D nature of the perovskite film. With the blue shift of EL spectra, the corresponding CIE value also changed from the sky blue to blue region, suggesting the successful achievement of a blue-emissive perovskite LED.

### Modulation of recombination zone position

**Fig. 4:**
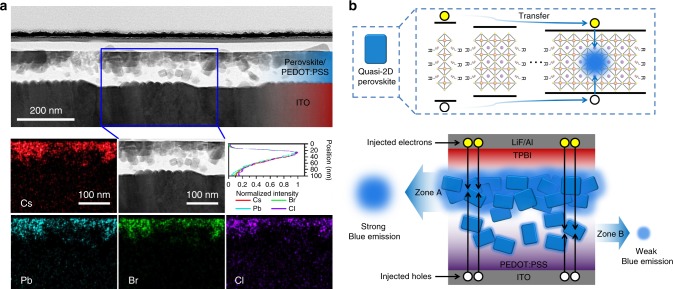
Vertical morphology of the perovskite thin film and schematic diagram of the modulation of recombination zone position. **a** Cross-section scanning transmission electron microscope (STEM) image of a indium tin oxide (ITO)/PEDOT:PSS/perovskite film. Energy-dispersive spectrometer (EDS) measurement was also used at the region marked with the blue lines to illustrate the elemental distribution of Cs, Pb, Br, and Cl. The position versus normalized intensity curves illustrate the integrated intensity of the Cs, Pb, Br, and Cl signals as a function of depth. The perfect overlapping suggests the homogenous distribution of all four signals in perovskite crystals. **b** The top schematic diagram shows that each perovskite crystal is in a quasi-2D structure, and energy transfer and/or charge transfer occurs after the injection of electrons and holes. The bottom schematic diagram shows that as most of the perovskite crystals disperse at the top of the film, if the injected electrons and holes can recombine in Zone A, a strong blue emission can be obtained; however, if the injected electrons and holes can only recombine in Zone B, the film shows a weak blue emission

Recently, several reports have shown that a vertically non-uniform distribution of perovskite nanostructure exists in quasi-2D perovskite thin films. Wang et al.^36^ reported a vertical element distribution in the perovskite multi-quantum wells in which the Pb signal gradually decreased from the top to the bottom of the perovskite thin film, suggesting that the 3D perovskite was mainly located at the top of the perovskite film, whereas the 2D perovskite was enriched at the bottom. In addition, Shang et al.^25^ reported that only the PL from 3D perovskite could be detected under photoexcitation from the top side, whereas PL from the 2D perovskite appeared with photoexcitation from the bottom side, indirectly reflecting the vertical distribution of the perovskite nanostructure. According to these previous studies, it can be expected that the position of the recombination zone for the injected charge carriers is critical to achieving a highly efficient perovskite LED if a vertically non-uniform distribution of nanostructure is existed in the perovskite thin film. Therefore, we used a scanning transmission electron microscope to study the vertical morphology of the perovskite thin film to reveal the distribution of the perovskite. According to Fig. 4a, we obtained a PEDOT:PSS/perovskite hybrid film without a distinct interface, although both the PEDOT:PSS and perovskite films were deposited layer by layer. The majority of perovskite crystals dispersed at the top of the hybrid film, whereas some crystals were embedded within the PEDOT:PSS layer, leaving a perovskite capping layer above the PEDOT:PSS film and resulting in a vertically non-uniform distribution of the perovskite crystals. We attributed this result to the rough PEDOT:PSS films regardless of the film thickness (Supplementary Figure 7), which allowed a few perovskite crystals filling into the voids and being embedded by the PEDOT:PSS layer.

In addition, we also used an energy-dispersive spectrometer (EDS) to further unveil the elemental distribution of perovskite crystals. Figure 4a shows the distribution of the Cs, Pb, Br, and Cl signals and their integrated signals as a function of depth. It is obvious to find that most of the elements present at the top of the film, showing a similar distribution without distinct compositional difference. In addition, the integrated Cs, Pb, Br, and Cl signals perfectly overlap with each other as a function of depth, illustrating that all of the perovskite crystals (from top to bottom) are in a similar stoichiometric ratio. This result provides strong evidence that every single perovskite crystal has a similar quasi-2D structure and that no obvious nanostructural distribution occurred in our case, which is different from that observed in previously mentioned reports^25,36^. Therefore, each perovskite crystal is a single emitter and by combining the EL spectra of the CsPbCl_0.9_Br_2.1_ perovskite LEDs with different ratios of PEABr, we can conclude that in a working perovskite LED, highly efficient energy transfer and/or charge transfer occurs within those single quasi-2D perovskite crystals, as illustrated in the top schematic diagram in Fig. 4b. In addition, the observation of vertically non-uniform distribution of the perovskite crystals encouraged us to further modulate the position of the recombination zone in the device aiming to produce a more efficient perovskite LED.

The recombination zone is the region in which injected electrons and holes meet and recombine with each other. For LED applications, we expect that the recombination zone should be located at the emissive layer to convert electrical energy into light. In addition, the recombination zone width is also a critical parameter, and it is calculated to be ca. 5.5 nm in our best perovskite LED (Supplementary Note 1 and Supplementary Figure 8). As previously mentioned, the majority of emissive perovskite crystals dispersed on the top of the perovskite film (assigned Zone A in Fig. 4b), while a small number of perovskite crystals dispersed at the bottom of the perovskite film (assigned Zone B in Fig. 4b). Therefore, it should be much better for the injected electrons and holes to recombine in Zone A rather than in Zone B to activate the greatest number of perovskite crystals to realize a strong blue emission, as illustrated in the bottom schematic diagram of Fig. 4b.

**Fig. 5:**
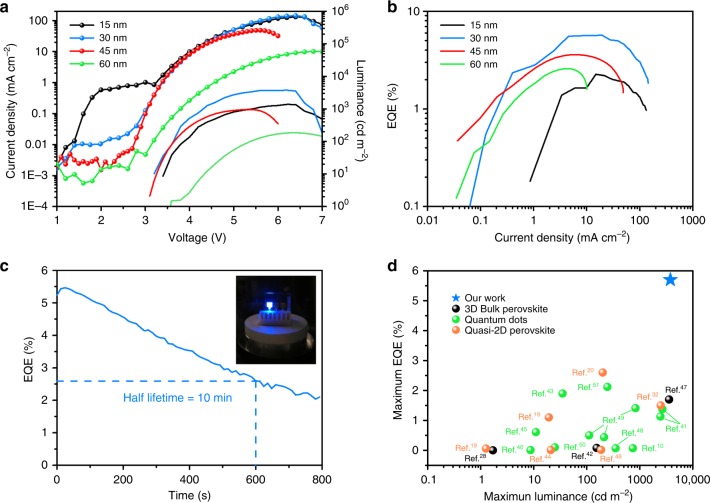
Performance of perovskite light-emitting diode (LEDs) (100% phenylethylammonium bromide (PEABr) case) with different PEDOT:PSS thicknesses. **a** Characterization of current density and luminance versus voltage. The solid lines and lines with dots correspond to luminance and current density, respectively. **b** Characterization of external quantum efficiency (EQE) versus current density. **c** Operating lifetime of a perovskite LED under a constant applied voltage of 4.4V and a photo of a working perovskite LED. **d** Comparison of our work with the currently published results based on blue-emissive perovskite LEDs with emission in the range of 450–500 nm

Generally, the position of the recombination zone in the emissive layer can be simply controlled by the thickness of the hole and electron injection layers^37–39^. Considering perovskite LED is typically a hole-dominant device when using PEDOT:PSS and TPBi as the hole and electron injection layer, respectively, we focused our study on the thickness control of the PEDOT:PSS layer to obtain an optimized position of the recombination zone^35^. We tuned the thickness of the PEDOT:PSS layer from 15 to 60 nm in the CsPbCl_0.9_Br_2.1_ perovskite LEDs with 100% PEABr, which has shown the best EQE in our study. According to Fig. 5a, b and Table 2, when the PEDOT:PSS layer was thick (60 nm), a relatively poor EQE of 2.6% was obtained in the perovskite LED because in a thick PEDOT:PSS case, the injected holes should take a relatively long time to pass through the thick PEDOT:PSS layer and subsequently inject into the valence band of perovskite, which results in a recombination zone position closer to Zone B. When we decreased the film thickness from 60 to 45 nm and 30 nm, the EQE of the perovskite LEDs increased dramatically from 2.6% to 3.6% and 5.7%, respectively. In such a case, the thinner PEDOT:PSS layer allowed the injected holes to pass through in a shorter time, resulting in a position shift of the recombination zone toward Zone A, which the electrons can reach in a short time. As the position of the recombination zone gradually shifted from Zone B to Zone A as the PEDOT:PSS thickness decreased, more and more perovskite crystals were activated, leading to an increase in device performance. However, when we further decreased the PEDOT:PSS thickness to 15 nm, the perovskite LED showed a relatively poor EQE of 2.2%. This result can be attributed to the poor coverage of such an ultra-thin PEDOT:PSS film on ITO, which may allow the perovskite crystals to directly get contact with the bottom electrode and therefore led to a severe leakage current in the perovskite LED (as shown in Fig. 5a) that degraded device performance. It is worth emphasizing that based on recombination zone modulation, our best blue perovskite LED achieved a record luminance of 3780 cd m^−2^ and a record EQE of 5.7%, which is more than twice that of the currently published data on blue perovskite LEDs, as shown in Fig. 5d^10,19,20,28,32,40–51^. Our devices also obtained excellent device reproducibility with average EQE of 4.5% (Supplementary Figure 9).

**Table 2:** Performance of perovskite LEDs (100% PEABr case) with different PEDOT:PSS thicknesses

PEDOT:PSS Thickness	*V*_T_ (V)	EL peak (nm)	Max. L. (cd m^−2^)	Max. CE (cd A^−1^)	Max. EQE (%)	FWHM (nm)	CIE
15 nm	3.4	481	1380	2.3	2.2	21	(0.106,0.194)
30 nm	3.2	480	3780	6.1	5.7	21	(0.102,0.178)
45 nm	3.1	480	965	3.9	3.6	21	(0.101,0.155)
60 nm	3.6	480	178	2.6	2.6	22	(0.101,0.148)

*LED* light-emitting diode, *PEDOT:PSS* Poly(3,4-ethylenedioxythiophene):poly(styrene sulfonate), *CE* current efficiency, *EQE* external quantum efficiency, *PEABr* phenylethylammonium bromide

In addition, our devices presented decent spectrum stability with no shift of the EL spectrum observed when we increased the applied voltage of the LED from 3.6 to 5.6 V (Supplementary Figure 10a). However, when we further increased the applied voltage exceeding 6 V, the EQE and luminance of the device started to drop, and the EL spectrum also red shifted slightly by around 8 nm, which could be attributed to the phase separation within the perovskite film due to the migration of Cl^−^ and Br^−^^40^. Moreover, the operating lifetime of the devices were also studied under a constant applied voltage of 4.4 V, as shown in Fig. 5c. Our device obtained a half lifetime of around 10 min, which is one of the best results among the reported lifetimes of blue-emitting perovskite LEDs, as summarized in Supplementary Table 1. In addition, the EL spectrum of the device was tracked during the operation. As shown in Supplementary Figure 10b, the initial emission peak was located at 480 nm and no spectrum shift was observed at the initial 1 min, followed by a slight red shift of 3 nm in the next 3 min. During the operating time from 3 to 9 min, the emission peak continuously shifted from 483 to 488 nm and eventually pined at 488 nm for the rest of testing time. We attributed this slight red shift of spectrum to the possible phase separation occurred in the mixed-halide perovskite system. It is worth noting that other degradation processes may also occur, which requires a further investigation in the future to fully unveil the degradation mechanism.

## Discussion

Here, we demonstrate a highly efficient blue perovskite LED by combining the strategies of composition engineering in quasi-2D perovskite crystals and modulation of the recombination zone position. In terms of composition engineering, CsCl, CsBr, and PbBr_2_ are used to prepare the precursor solution, and the molar ratio of CsCl:CsBr:PbBr_2_ of 9:1:10 is used to form the blue-emissive 3D perovskite (CsPbCl_0.9_Br_2.1_) without solubility problem. Subsequently, PEABr is introduced into the CsPbCl_0.9_Br_2.1_ perovskite to passivate the trap states and form quasi-2D perovskite, which helps to obtain a blue-emissive quasi-2D perovskite thin film with PLQY up to 27%. Moreover, we observe a vertically non-uniform distribution of quasi-2D perovskite crystals in the PEDOT:PSS/perovskite hybrid film in which the majority of perovskite crystals disperse on top of the film and only a few are embedded within the PEDOT:PSS layer. Therefore, we modulate the position of the recombination zone to the top of the perovskite film by simply controlling the thickness of the PEDOT:PSS layer to allow the majority of charges recombine in the perovskite emitters. With this strategy, we successfully demonstrate an efficient blue perovskite LED with record EQE of 5.7% and luminance of 3780 cd m^−2^. The EL peak is located at 480 nm with FWHM of 21 nm. Our study shows that it is possible to further improve the performance of blue perovskite LEDs by carefully engineering the composition and dimension of the perovskite crystals, as well as the recombination zone position in the devices, paving the way for realization of full-color displays and white-light illumination with perovskite LEDs. However, the lifetimes of our devices are still far from that of commercial LEDs, and it is one of the tough issues has to be solved in perovskite LED field. Our finding indicates that phase separation is probably the main issue causing the device degradation in a mixed-halide perovskite system. Therefore, a pure-halide perovskite system with pure blue and red emission may be ultimately needed for stable perovskite LEDs. In addition, heat produced by the injected current and photon reabsorption may also cause a thermal stability issue (change of film morphology or degradation of perovskite component, etc.). In such case, an efficient device structure design with excellent heat radiation and light out coupling needs to be developed. Moreover, considering the ionic nature of perovskite crystals, the strong electric field in a thin film perovskite LED also could be another possible factor for device degradation, and this would be more severe for a blue perovskite LED as which needs higher applied voltage for operation. The real degradation mechanism of perovskite LEDs is still unclear in this field, and it is of great necessity for an intelligent design for stable perovskite LEDs.

## Methods

### Materials and chemicals

CsBr (99.999%), CsCl (99.999%), and PbBr_2_ (99.999%) were purchased from Sigma-Aldrich. PEABr (99.5%) was purchased from Xi’an Polymer Light Technology Corp. DMSO (>99.0%) was purchased from TCI. LiF was purchased from Alfa Aesar. PEDOT:PSS (CH 8000) was purchased from Heraeus. TPBi was purchased from Lumtec. All chemicals were used as received.

### Perovskite solution preparation

The CsPbCl_*x*_Br_3 − *x*_ precursor solutions were prepared by dissolving appropriately stoichiometric CsCl, CsBr, and PbBr_2_ in DMSO under continuous stirring for 12 h at room temperature, keeping the molar concentration of Pb^2+^ at 0.2 M. For the quasi-2D perovskite precursor solution, 0.02 mmol CsBr, 0.18 mmol CsCl, 0.2 mmol PbBr_2_, and an appropriate amount of PEABr were dissolved in 1 mL DMSO under continuous stirring for 12 h at room temperature. The ratio of *x*% PEABr refers to the molar ratio between PEABr and PbBr_2_ (i.e., *m*_PEABr_/*m*_PbBr2_ = *x*%).

### Perovskite LED fabrication

The patterned ITO-coated glass substrates were subsequently cleaned by sonication in detergent, deionized water, acetone, and isopropyl alcohol and then dried at 65 °C in a baking oven. After a 4-min oxygen plasma treatment for ITO, PEDOT:PSS (CH 8000) aqueous solution was spin-coated onto the ITO substrate at 3500 rpm for 50 s and baked at 150 °C for 15 min in ambient air. The thickness of the PEDOT:PSS was tuned by diluting the stock solution with different amounts of deionized water. Perovskite precursor solution was then spin-coated onto the PEDOT:PSS film at 2500 rpm for 2 min in an N_2_-filled glovebox. Subsequently, the film was annealed at an optimized temperature of 60 °C for 5 min (Supplementary Figure 11). Finally, TPBi (40 nm) and LiF/Al electrodes (1 nm/100 nm) were deposited using a thermal evaporation system under a high vacuum of <1 × 10^–6^ torr. The device active area was 10 mm^2^ as defined by the overlapping area of the ITO and Al electrodes.

### Perovskite film and device characterizations

The film thicknesses were determined with a Dektak 150 stylus surface profiling system (Veeco). The ultraviolet–visible absorption spectra were recorded on an HP 8453E spectrophotometer. Steady-state PL was recorded using a Horiba Fluorolog system equipped with a single grating and a monochromatized Xe lamp was used as the excitation source. The crystalline structure of the perovskite films was investigated with an X-ray diffractometer (PANalytical X’pert PRO) equipped with a Cu-Kα X-ray tube, using ITO/PEDOT:PSS/perovskite as samples. The SEM images were obtained with a Zeiss EVO 18 SEM based on the sample structure of ITO/PEDOT:PSS/perovskite. The trap densities of the perovskite films were extracted by the dark current–voltage characteristics of the hole-only devices in the device architecture of the ITO/PEDOT:PSS/perovskite/MoO_3_ (10 nm)/Ag (100 nm) through a computer-controlled Keithley 2400 source meter. PLQYs of the perovskite films were recorded by a commercialized PLQY measurement system from Ocean Optics with excitation from a 365-nm LED. Transient PL decay spectra were measured using Quantaurus-Tau fluorescence lifetime measurement system with excitation wavelength of 365 nm (C11367-03, Hamamatsu Photonics Co., Japan). UPS spectra were collected on Thermo Fisher Scientific Escalab 250 Xi equipment with an applied bias of −5 V. A He I ultraviolet radiation source (21.22 eV) was used. The pressure in the analysis chamber during measurement was about 5 × 10^−10^ mbar. The energy scale was based on the Fermi edge of gold (0 eV). The cross-sectional STEM images and EDS measurement were taken with a FIB-TEM Helios Nanolab 450S. The atomic force microscope measurements were carried out using a Digital Instrumental Multimode Nanoscope IIIa in tapping mode. Capacitance–voltage measurements were carried out with a Paios 4.0 Measurement Instrument (FLUXiM AG, Switzerland). The I–V–L curve, EL spectrum, EQE, and operating lifetime of the perovskite LED were recorded simultaneously by a commercialized system (XPQY-EQE-350-1100, Guangzhou Xi Pu Optoelectronics Technology Co., Ltd.) that was equipped with an integrated sphere (GPS-4P-SL, Labsphere) and a photodetector array (S7031-1006, Hamamatsu Photonics). All of the device characterization tests of the perovskite LEDs were carried out in an N_2_-filled glovebox.

## Supplementary information

Supplementary Information

## Data Availability

The data that support the findings of this study are available from the corresponding author upon reasonable request.
