# Supplementary material for: Modulation of recombination zone position for quasi-two-dimensional blue perovskite light-emitting diodes with efficiency exceeding 5%
Source: Nat Commun. 2019 Mar 4;10:1027. doi: 10.1038/s41467-019-09011-5 (PMC6399279; doi:10.1038/s41467-019-09011-5)
Supplement: Supplementary file 1 — Supplementary Information [file 41467_2019_9011_MOESM1_ESM.pdf]

Supplementary Information

Modulation of Recombination Zone Position for Quasi-Two-Dimensional Blue Perovskite Light-Emitting Diodes with Efficiency Exceeding 5%

Li et al.

Supplementary Figures

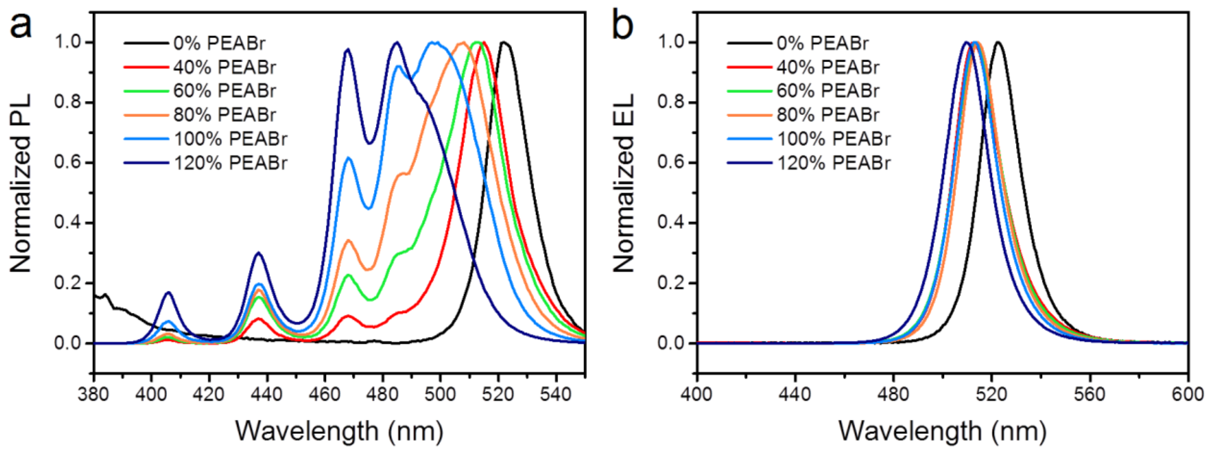

Supplementary Figure 1 | Emission spectra of $CsPbBr_3$ perovskites with various ratios of PEABr. **a**, PL spectra of perovskite thin films. The excitonic emission peaks of 406 nm, 437 nm, 468 nm and 485 nm illustrate the formation of 2D perovskite and correspond to n=1, 2, 3 and 4 2D perovskite, respectively. **b**, EL spectra of perovskite LEDs. It is obvious that no EL emission from 2D perovskite (n=1, 2, 3 and 4) can be detected because a highly efficient energy transfer and/or charge transfer existed in this system that retarded the achievement of blue emission in perovskite LEDs.

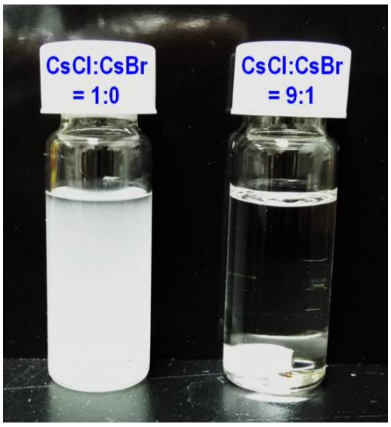

Supplementary Figure 2 | Photos of $CsPbCl_xBr_{3-x}$ precursor solutions. The precipitation in the left solution shows the solubility issue that occurred when we further increased the ratio of CsCl:CsBr from 9:1 to 1:0 in the precursor solution.

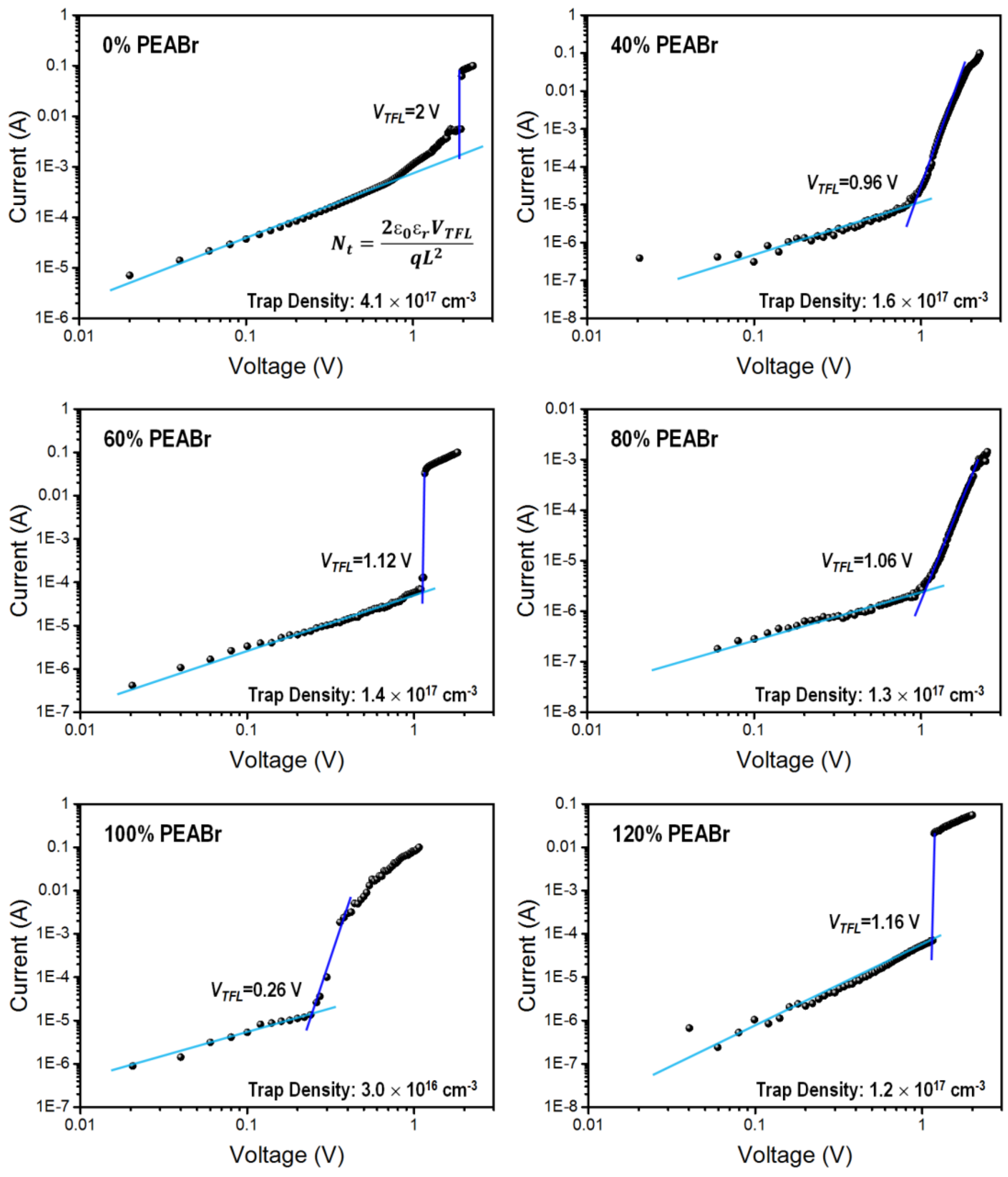

Supplementary Figure 3 | Trap density extraction by dark current-voltage measurement of the hole-only device, with the device structure of ITO/PEDOT:PSS/perovskite/MoO_3/Ag. The light-blue lines represent the ohmic regime of each case, and the dark-blue lines indicate the trap-filled limit (TFL) regime with the onset voltage (V_{TFL}). The trap density (N_t) of $CsPbCl_{0.9}Br_{2.1}$ thin films with different ratios of PEABr can be extracted by the equation inserted in the first figure, where ε_0, q and L represent vacuum permittivity, elementary charge and thickness of the perovskite film, respectively; ε_r is the average relative dielectric constant of $CsPbCl_{0.9}Br_{2.1}$ thin films with different ratios of PEABr extracted from the capacitance-voltage curve as shown in Supplementary Figure 4.

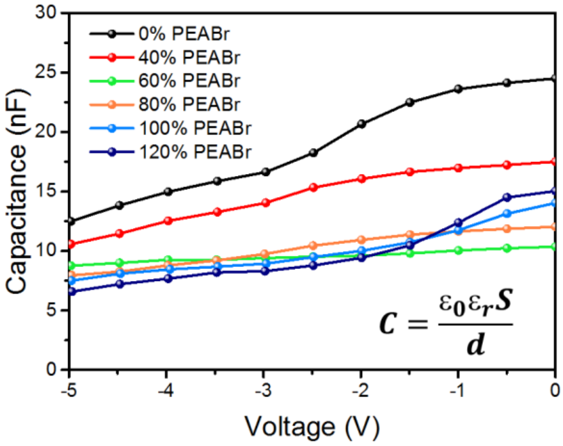

PEABr Ratio	ε_r
0%	15
40%	12.5
60%	9.5
80%	8.9
100%	8.4
120%	7.6

Supplementary Figure 4 | Relative dielectric constant (ε_r) extraction by capacitance (*C*) – voltage (*V*) measurement, with the device structure of ITO/PEDOT:PSS/perovskite/Ca/Al. By confirming the *C* value of $CsPbCl_{0.9}Br_{2.1}$ thin films with different ratios of PEABr by the saturated part in the *C*-*V* curves towards negative voltage, we extracted the ε_r value of each case by the inserted equation and summarized them in the table, where ε_0, *S* and *d* represent vacuum permittivity, device area and thickness of the perovskite film, respectively. The decrease of ε_r with the increasing amounts of PEABr can be attributed to the insulating nature of PEABr and dimensional change in perovskite from 3D to 2D.

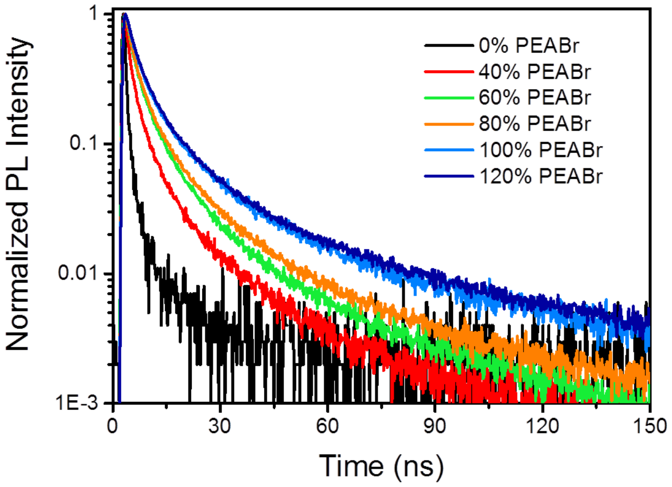

Supplementary Figure 5 | PL lifetime measurement of $CsPbCl_{0.9}Br_{2.1}$ perovskites with various concentrations of PEABr. The average lifetime of 100%-PEABr case is 22.58 ns.

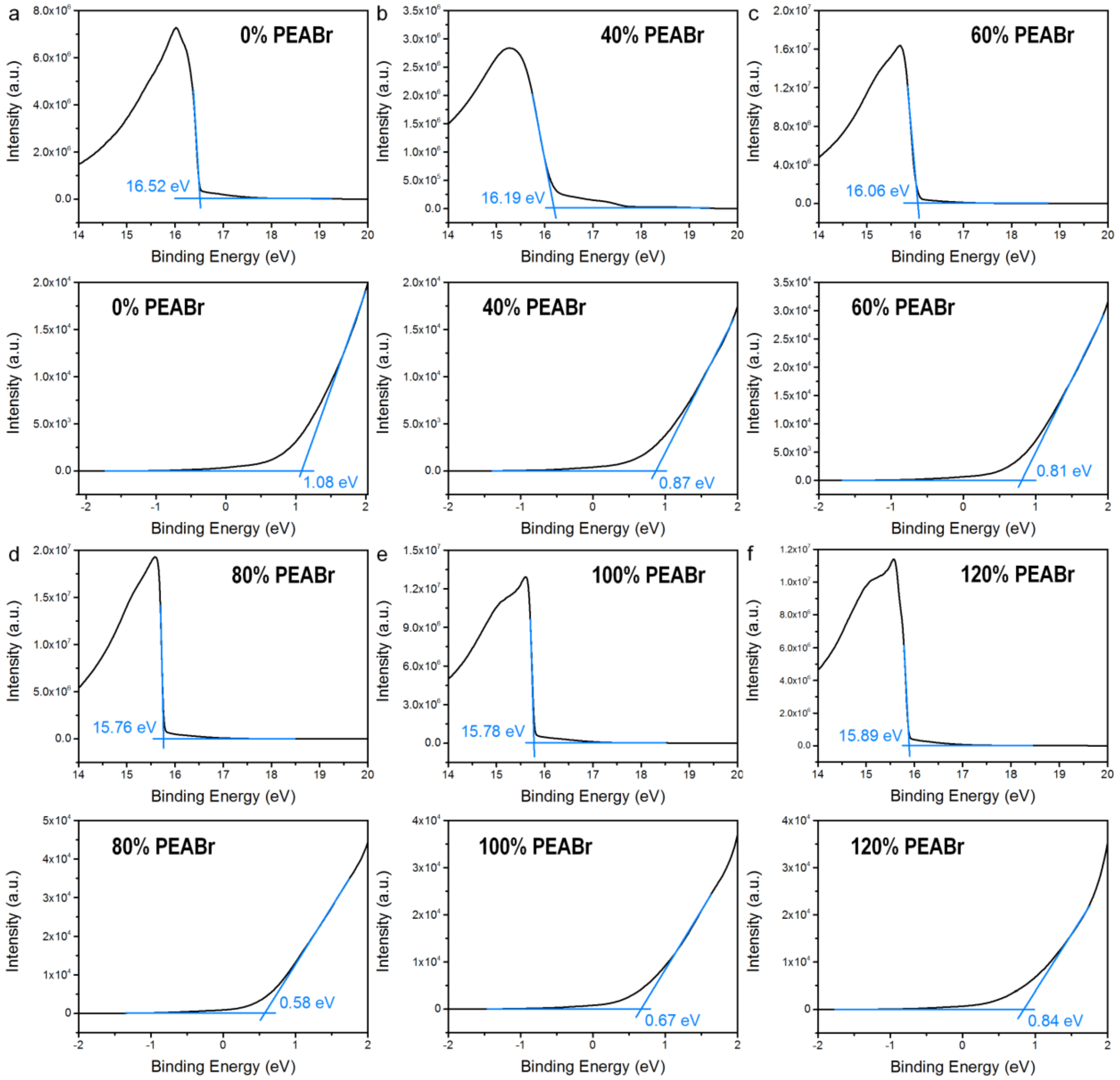

Supplementary Figure 6 | UPS spectra of $CsPbCl_{0.9}Br_{2.1}$ perovskites with various concentrations of PEABr. (**a**) 0% PEABr. (**b**) 40% PEABr. (**c**) 60% PEABr. (**d**) 80% PEABr. (**e**) 100% PEABr. (**f**) 120% PEABr. In the UPS measurements, a He I source with photon energy of 21.22 eV was used to excite the sample. Therefore, the calculated valance bands were 5.78 eV, 5.90 eV, 5.97 eV, 6.04 eV, 6.11 eV and 6.17 eV for 0% PEABr, 40% PEABr, 60% PEABr, 80% PEABr, 100% PEABr and 120% PEABr samples, respectively.

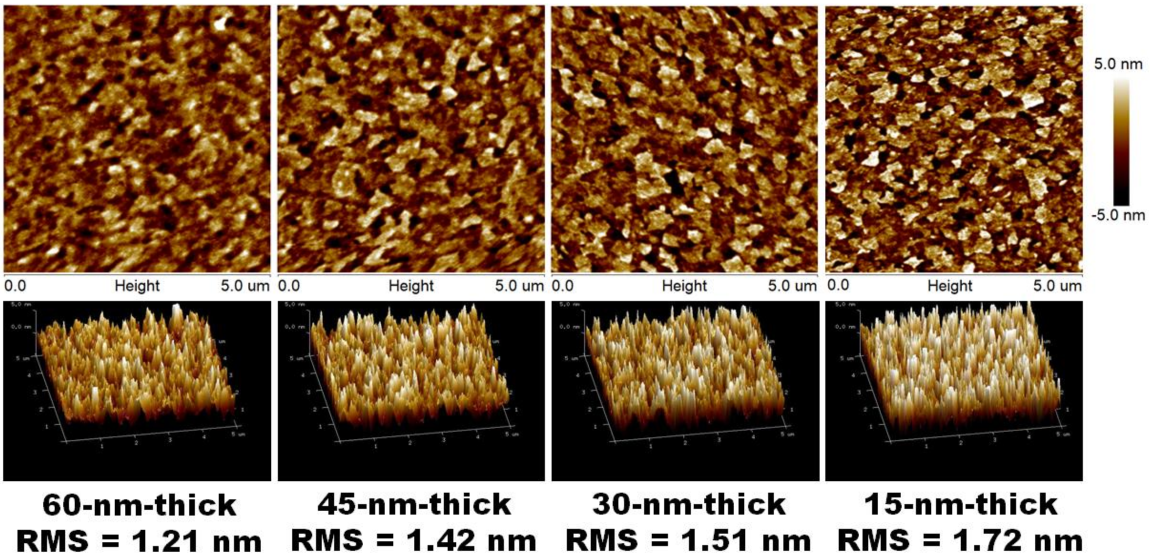

Supplementary Figure 7 | Atomic force microscope images of PEDOT:PSS (CH8000) films with different thicknesses. A few perovskite crystals are likely to fill in the voids and form a vertically non-uniform distribution.

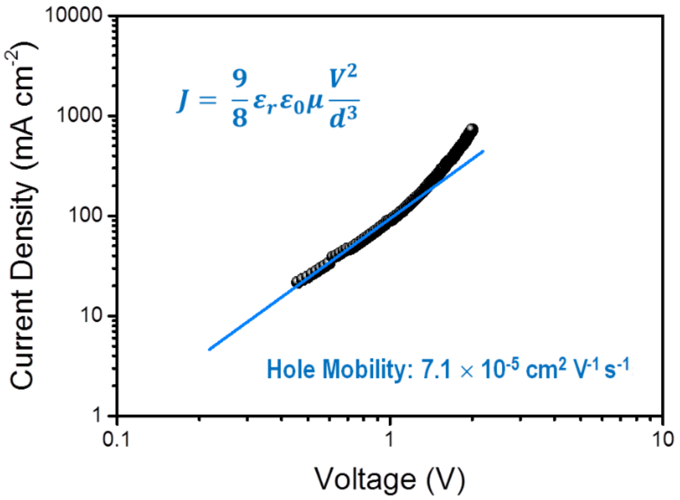

Supplementary Figure 8 | Hole mobility measurement by SCLC method, with the device structure of ITO/PEDOT:PSS/perovskite/MoO_3/Ag. The mobility was determined by fitting the dark current to the model of a single-carrier SCLC, which is described by the inserted equation, where J is the current density, μ is the mobility, ε_0 is the permittivity of free space, ε_r is the relative permittivity of the material, d is the thickness of the active layer, and V is the effective voltage. The effective voltage was obtained by subtracting the built-in voltage (V_{bi}) and the voltage drop (V_s) from the series resistance of the whole device except for the active layers from the applied voltage (V_{appl}), i.e. $V = V_{appl} - V_{bi} - V_s$. The mobility can be calculated from the slope of the $J^{1/2}$-V curve.

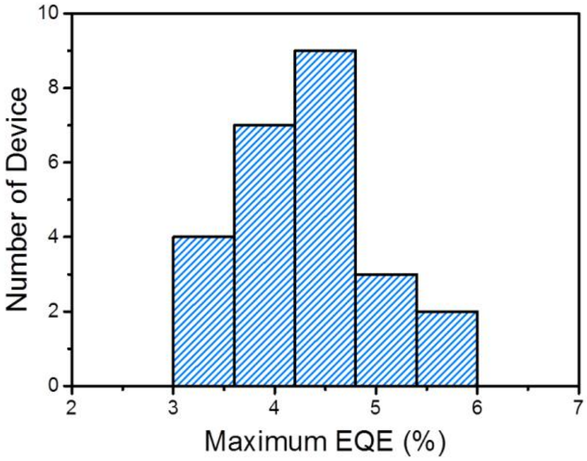

Supplementary Figure 9 | Statistical performance of $CsPbCl_{0.9}Br_{2.1}$ perovskite LEDs with 100% PEABr. The average EQE of 25 devices was 4.5% with a relative standard deviation of 16%.

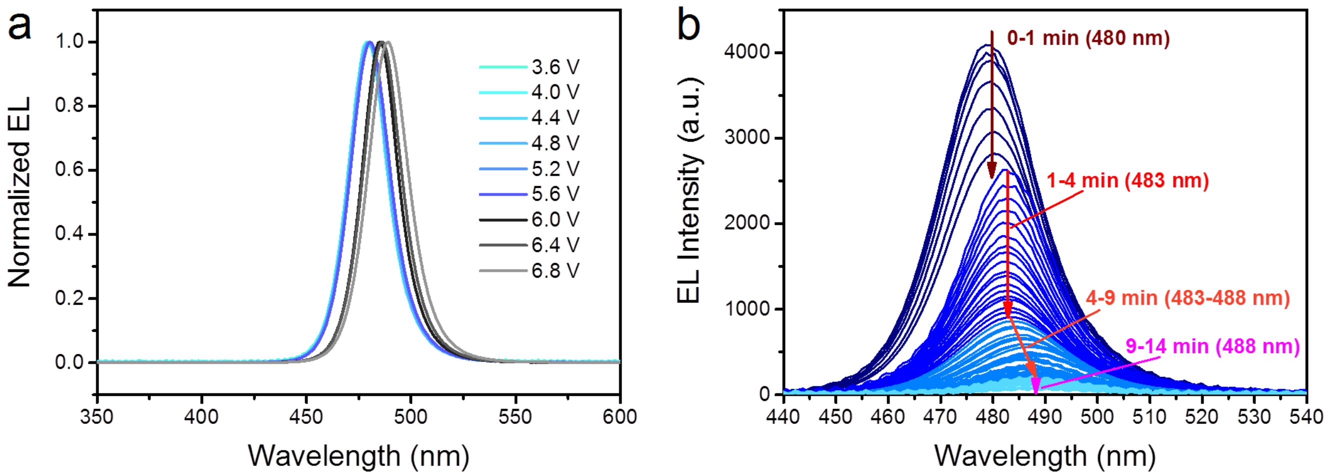

Supplementary Figure 10 | Spectrum stability of $CsPbCl_{0.9}Br_{2.1}$ perovskite LED with 100% PEABr. a, Initial EL spectrum under different applied voltages. **b**, EL spectrum under a constant applied voltage of 4.4 V as a function of time. The EL peak located at 480 nm in the first 1 min, followed by a shift to 483 nm in the next 3 min. Subsequently, during the operating time from 4-9 min, the EL peak gradually shifted from 483 to 488 nm, and maintained the 488-nm peak for the rest of the testing time. This peak shift process illustrates that the phase separation in perovskite films actually undergoes different processes.

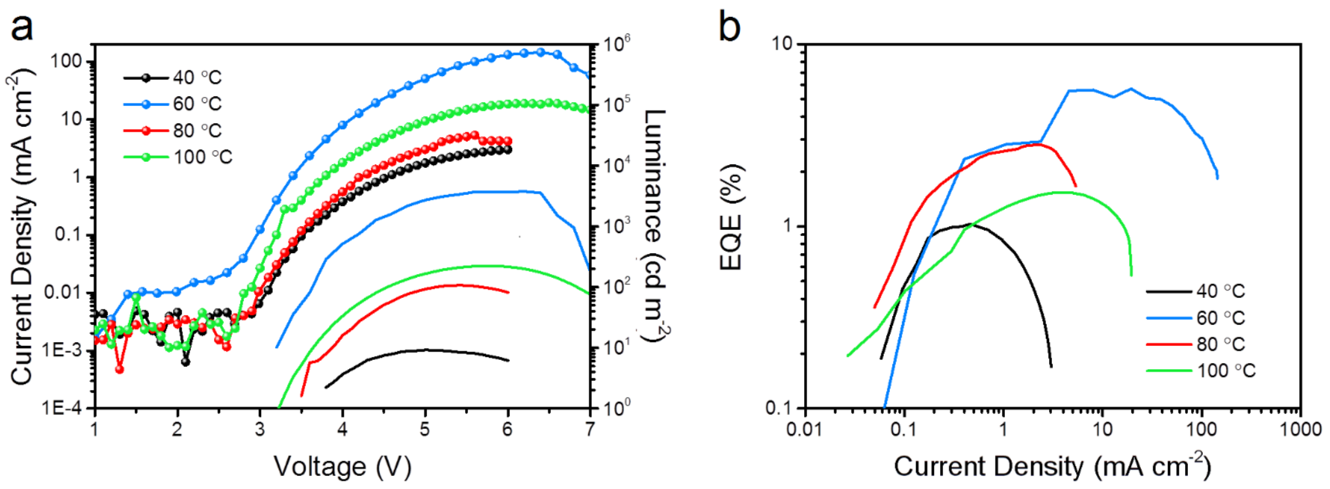

Supplementary Figure 11 | Performance of perovskite LEDs (100% PEABr case) with different annealing temperatures. a, Characterization of current density and luminance versus voltage. The solid lines and lines with dots correspond to luminance and current density, respectively. **b**, Characterization of EQE versus current density. The perovskite LED annealed at 60 ℃ shows the best EQE and luminance, illustrating that 60 ℃ is the optimized annealing temperature for our perovskite films.

Supplementary Table

Supplementary Table 1 | Lifetime summary of our work and previously published results about the blue perovskite LEDs.

Composition	Testing Condition	Half Lifetime	Reference
$MAPbBr_{1.5}Cl_{1.5}$	Constant Voltage: 6 V	30 s	[1]
$CsPbBr_xCl_{3-x}$ nanocrystals	Constant Current Density:10 mA cm^{-2}	~5 s	[2]
$IPA/PEA_2MA/Cs_{n-1}Pb_nBr_{3n+1}$	Initial Luminance: 10 cd m^{-2}	10 min	[3]
	Initial Luminance: 20 cd m^{-2}	4 min	
	Initial Luminance: 210 cd m^{-2}	0.5 min	
$CsPb_{1-y}Mn_yCl_{3-x}Br_x$	Constant Current Density:1 mA cm^{-2}	~15 s	[4]
$CsPbCl_{0.9}Br_{2.1}$ + 100% PEABr	Constant Voltage: 4.4 V	10 min	This work

Supplementary Note 1

The calculation of recombination zone width.

The recombination zone width (W) is defined as the average path the carrier traverses during the recombination time and it can be quantitatively described by the following equation[5]:

$$W = \mu_{\mathrm{eff}} \times F \times \tau_{\mathrm{rec}} \quad (1)$$

where $\mu_{\mathrm{eff}} = \mu_{\mathrm{h}} + \mu_{\mathrm{e}}$ is the effective mobility of mobile holes (μ_{h}) and electrons (μ_{e}), while F and τ_{rec} represent the electric field intensity within the emissive layer and the recombination lifetime of the charge carriers, respectively. In Cs-based 2D perovskite using PEABr as the organic spacer, the hole mobility is 2 to 3 order higher than its electron mobility[6]. Therefore, the μ_{eff} (effective mobility) approximately equalled to μ_{h} (hole mobility) in this case. The hole mobility of our quasi-2D perovskite ($CsPbCl_{0.9}Br_{2.1}$ with 100% PEABr) is extracted by the space-charge limited current (SCLC) measurement, as shown in Supplementary Figure 8. For an easy calculation, the F is assigned as the average electric field intensity $F_{\mathrm{ave}} = U/d$ of the whole device, where U is the applied voltage and d is the device thickness. We choose U of 4.4 V and d of 130 nm here, as at such voltage the device reaches the best EQE. In addition, in reality, the τ_{rec} is related to the intensity of electric field ($\tau_{\mathrm{rec}}(F)$) as a high electric field could cause a faster recombination (or quenching) of the charge carriers. In our case, for an easy calculation, the τ_{rec} is assigned as the average lifetime (τ_{ave}) of charge carriers extracted from the PL lifetime (22.58 ns) shown in Supplementary Figure 5. The actual lifetime of charge carriers in the device should be shorter than this value because of the presence of electric field within the device, therefore, the calculated recombination zone W here represents its possible upper limit. Based on the above analysis, the calculated recombination zone width is ca. 5.5 nm.

Supplementary References

[1] Wang, Z., Cheng, T., Wang, F., Dai, S. & Tan, Z. Morphology engineering for high-performance and multicolored perovskite light-emitting diodes with simple device structures. *Small* **12,** 4412–4420 (2016).

[2] Gangishetty, M. K., Hou, S., Quan, Q. & Congreve, D. N. Reducing architecture limitations for efficient blue perovskite light-emitting diodes. *Adv. Mater*. **30,** 1706226 (2018).

[3] Xing, J. et al. Color-stable highly luminescent sky-blue perovskite light-emitting diodes. *Nat. Commun.* **9,** 3541 (2018).

[4] Hou, S., Gangishetty, M. K., Quan, Q. & Congreve, D. N. Efficient blue and white perovskite light-emitting diodes via manganese doping. *Joule* **2,** 2421-2433 (2018).

[5] Kalinowski, J., Picciolo, L. C., Murata, H. & Kafafi, Z. H. Effect of emitter disorder on the recombination zone and the quantum yield of organic electroluminescent diodes. *J. Appl. Phys.* **89,** 1866 (2001).

[6] Yu, M. et al. Control of barrier width in perovskite multiple quantum wells for high performance green light–emitting diodes. *Adv. Optical Mater.* 1801575 (2018).
